# A classifier model for prostate cancer diagnosis using CNNs and transfer learning with multi-parametric MRI

**DOI:** 10.3389/fonc.2023.1225490

**Published:** 2023-11-09

**Authors:** Mubashar Mehmood, Sadam Hussain Abbasi, Khursheed Aurangzeb, Muhammad Faran Majeed, Muhammad Shahid Anwar, Musaed Alhussein

**Affiliations:** ^1^ Department of Computer Science, COMSATS Institute of Information Technology, Islamabad, Pakistan; ^2^ Department of Computer Science, Kohsar University Murree, Punjab, Pakistan; ^3^ Department of Computer Engineering, College of Computer and Information Sciences, King Saud University, Riyadh, Saudi Arabia; ^4^ Department of AI and Software, Gachon University, Seongnam, Republic of Korea

**Keywords:** transfer learning, convolutional neural network, deep learning, PCA, MRI images

## Abstract

Prostate cancer (PCa) is a major global concern, particularly for men, emphasizing the urgency of early detection to reduce mortality. As the second leading cause of cancer-related male deaths worldwide, precise and efficient diagnostic methods are crucial. Due to high and multiresolution MRI in PCa, computer-aided diagnostic (CAD) methods have emerged to assist radiologists in identifying anomalies. However, the rapid advancement of medical technology has led to the adoption of deep learning methods. These techniques enhance diagnostic efficiency, reduce observer variability, and consistently outperform traditional approaches. Resource constraints that can distinguish whether a cancer is aggressive or not is a significant problem in PCa treatment. This study aims to identify PCa using MRI images by combining deep learning and transfer learning (TL). Researchers have explored numerous CNN-based Deep Learning methods for classifying MRI images related to PCa. In this study, we have developed an approach for the classification of PCa using transfer learning on a limited number of images to achieve high performance and help radiologists instantly identify PCa. The proposed methodology adopts the EfficientNet architecture, pre-trained on the ImageNet dataset, and incorporates three branches for feature extraction from different MRI sequences. The extracted features are then combined, significantly enhancing the model’s ability to distinguish MRI images accurately. Our model demonstrated remarkable results in classifying prostate cancer, achieving an accuracy rate of 88.89%. Furthermore, comparative results indicate that our approach achieve higher accuracy than both traditional hand-crafted feature techniques and existing deep learning techniques in PCa classification. The proposed methodology can learn more distinctive features in prostate images and correctly identify cancer.

## 1 Introduction

A major challenge for medical science is cancer, which is the most widespread disease in humans around the globe. Cancer cells exhibit aggressive growth rates, and their precise diagnosis is pivotal to a patient’s survival. The most prevalent cancer diagnosed in men worldwide is PCa. Alarming statistics from the American Cancer Society predict approximately 288,300 new PCa cases in the United States by 2023, with an estimated 34,700 fatalities cancer society (1).

The conventional method for PCa classification relies on the Gleason Score (GS), as determined by analyzing biopsy samples. However, this conventional biopsy method has been found to exhibit reduced sensitivity in accurately identifying PCa. Furthermore, the Gleason classification method encounters challenges stemming from variations in interpretation, encompassing discrepancies among different observers (interobserver variability) and inconsistencies within assessments made by the same observer (intraobserver variability). These variations can predominantly be attributed to the heavy reliance on human interpretation within the Gleason classification method ŞCheck that all equations and special characters are displayed correctly.erbănescu et al. (2). Recent advancements in mpMRI have emerged as a pivotal tool for assessing the risk of PCa and improving the grading and classification of PCa Oberlin et al. (3); Bardis et al. (4). High-grade PCa is frequently characterized by the presence of more densely packed structures which can be effectively identified through the utilization of advanced MRI-based machine learning techniques. These approaches enable the detection and assessment of high-grade PCa, thereby enhancing diagnostic precision and treatment planning for patients. This study aims to create an automated method for classifying PCa, aiding radiologists’ assessments.

The implementation of quantitative assessments of mpMRI offers radiologists a valuable, noninvasive tool to enhance their clinical decision-making processes. Furthermore, it helps mitigate the discrepancies that can arise due to differences between readers. With the growing interest in the integration of artificial intelligence (AI) with medical practice, empowered by enhanced computational capabilities and the emergence of new AI methodologies, there has been a surge in studies introducing CAD approaches. These systems leverage machine-learning and deep-learning approaches to detect and classify tumors in medical imagery, with a pronounced impact on PCa detection and classification Abbasi et al. (5). This study aims to build an automated PCa classification method, addressing the limitations of traditional GS and improving the diagnostic potential offered by mp-MRI. Early-stage PCa identification is extremely important and beneficial for treatments. Identification of PCa using MRI images improves the rate of early diagnosis and assists in building a Computer Aided Diagnostic (CAD) system Jin et al. (6); Reda et al. (7). A pivotal objective within CAD systems is the development of objective and reproducible metrics for automated analysis Cem Birbiri et al. (8). The continuous refinement of PCa classification techniques holds significant importance, particularly in distinguishing between low and high-grade cancers.

To identify PCa, several approaches have been proposed. Most of them used traditional machine-learning approaches, to classify images and train classifiers Bardis et al. (4); Monni et al. (9); Abbasi et al. (5); Gillies et al. (10); Fehr et al. (11); Vignati et al. (12); Liu et al. (13); Ullah et al. (14); Shahzad et al. (15); Laghari et al. (16); Sobecki et al. (17); Giannini et al. (18); Wang et al. (19); Schelb et al. (20); Wildeboer et al. (21); Wibmer et al. (22). Many of these approaches used features of low radiomics focused on previous clinical reports, which may not fully leverage the entire information within the MRI images Källén et al. (23). Moreover, unsupervised approaches were previously used to acquire features that may contain unnecessary information or may exclude essential clues. Deep learning approaches recently acquired great performance and are widely used in classifying and identification tasks of both medical applications Esteva et al. (24); Albarqouni et al. (25); Yuan and Meng (26) and natural images LeCun et al. (27). They can train classifiers and learn features jointly. Because deep learning techniques have enormous potential and success, the authors use them to classify PCa. Deep learning-based architectures have yielded remarkable results because of their capability to autonomously acquire and represent features Tsehay et al. (28). Compared to conventional approaches, CNN-based models such as Alexnet demonstrated improved performance Kiraly et al. (29). A challenge associated with architectures like this is the substantial data needed for effective training such as in Chen et al. (30). Using transfer learning is an easier way to handle this problem. In order to extract features and identify data from one domain into another, transfer learning employs training experience as a sort of knowledge sharing Le et al. (31). Good performance can be accomplished with small training images by using the transfer learning technique Wildeboer et al. (32); Zhong et al. (33) that applies models of pre-trained images from other datasets. Furthermore, various MRI sequences of PCa present different concerns, and it is important to consider an effective way to incorporate different details. By combining details derived from multi-parametric images, a descriptive representation of PCa may be gained Cem Birbiri et al. (8).

A multi-parametric MRI transfer learning (mp-TL) system to identify PCa is presented in this study. To obtain features from various MRI sequences, the proposed transfer learning model has three branches: ADC and T2w (sagittal, trans-axial). The features extracted from these categories are combined in the model. For this study, we aim to utilize transfer learning techniques leveraging a family of networks of pre-trained EfficientNet models for the classification of prostate images. Compound scaling is used in the recently proposed Efficient-Nets architecture to balance the network’s three dimensions (Depth, Height, and Width). The proposed method demonstrates good performance in effectively classifying PCa images, contributing to enhanced diagnosis. The improvement in classifying PCa techniques is necessary to distinguish low and high-grade cancer. There is a need for an efficient deep learning-based architecture that efficiently classifies PCa images. In the proposed methodology, the important step for the classification of MRI images is the pre-processing stage. Pre-processing is used to process the PCa MRI images, and then the classification and feature extraction of PCa images is performed using deep CNN models. The contribution of this study is based on the classification of images and results in comparison with existing approaches. These are a few of this study’s major contributions.

• The proposed approach here harnesses transfer learning to jointly analyze multiple MRI sequences, rather than focusing solely on a single MRI sequence. This enables us to extract more discriminative features, leading to a substantial enhancement in PCa classification.
• To demonstrate the model’s effectiveness, the authors evaluate the PCa dataset utilizing a diverse range of Efficient-Net Models, encompassing B0, B5, and B7.
• Multi-view ensemble approach is used for classifying multi-parametric MRI images.
• An Efficient-Net model with fine-tuning and an additional Global Average Pooling (GAP) layer at the model’s end, serves as a crucial component. This not only extracts vital information but also forwards it to the activation function for further processing.
• The proposed approach’s effectiveness is highlighted through extensive experimentation conducted on the PCa dataset.

## 2 Related work

Numerous studies have been carried out by researchers to predict prostate MRI imaging. The literature on MRI image classification encompasses a range of both deep-learning and machine-learning techniques. There are various PCa MRI datasets that can be used for classification tasks, such as prostatex, ACRIN, and I2CVB. However, accessibility to these datasets is often limited or incomplete for many researchers. In contrast, Prostatex is a publicly accessible dataset specifically intended for research purposes.

For the MRI imaging classification of PCa, Chen et al. (34) suggested a deep-learning method focused on classification. A deep convolutional neural network, such as InceptionV3 and VGG-16 underwent pre-training on the ImageNet dataset. Subsequently, the multi-parametric magnetic resonance imaging dataset was fine-tuned. Xu et al. (35) employed residual networks for the identifying PCa. ResNets have demonstrated a capacity to learn both low-level and high-level features, making them well-suited for detecting subtle and intricate patterns in medical images, which are often indicative of diseases like PCa.Their study showcased the feasibility of training residual networks to acquire features that are valuable for identifying suspicious indicative of PCa.

In this study, Alkadi et al. (36), the authors employ a deep convolutional neural network to segment prostate lesions in T2W MRI images. They introduce a 3D sliding window technique for 3D context while maintaining computational efficiency. The approach distinguishes cancerous and non-cancerous tissues, with comparable results to multi-parametric systems, avoiding intricate alignment steps. This comprehensive study Viswanath et al. (37) assesses the performance of supervised classifiers in a multisite approach for detecting prostate cancer (PCa) extent using T2w MRI. The primary focus is on radiomic features extracted from high-resolution T2 images. The aim is to enhance the accuracy and timeliness of diagnoses in the context of medical imaging, particularly for PCa detection, where early and precise identification is critical for effective treatment.

Authors in this study Muhammad et al. (38) have highlighted the potential of utilizing a combination of multiple parameters, either as individual parameters or integrated multiple parameters within a machine learning framework, to enhance diagnostic capabilities. Their Schelb et al. (20) study highlights the effectiveness of training deep learning models to recognize and segment lesions in T2 and diffusion MRI data, significantly improving the clinical evaluation of MRI data. The UNet model was trained using cross-validation, incorporating split-sample techniques, and subsequently validated using an external test set. Singh et al. (39) suggest the use of deep neural networks for cribriform pattern classification. In this study, the authors introduce an automated image classification system employing deep learning and hand-crafted features to analyze prostate images. The focus is on detecting cribriform patterns, with results demonstrating diagnostic potential.

With notable advancements in computer vision, particularly in target recognition and identification through deep convolutional neural networks, the medical imaging research community is increasingly delving into the exploration of diverse CNN architectures. These architectures offer substantial potential for enhancing the accuracy of cancer detection systems. In this study, Yoo et al. (40) developed and introduced an automated pipeline based on CNN. This pipeline is designed to analyze images on a per-patient basis, aiming to detect clinically relevant PCa.

Bulten et al. (41) reported that the implementation of a semi-automatic labeling system eliminated the need for pathologists to manually annotate the images. A high degree of agreement with the reference norm has been obtained by the established framework. The deep learning method outperformed pathologists in different observation trials. Li et al. (42) clarify that for the diagnosis of disease, histology analysis is also seen as the gold standard. By reducing test time and inter-observer variability, computer-aided diagnostic software can theoretically further optimize existing pathology workflows. Previous cancer grading analyses have predominantly focused on the classification of predefined regions of significance or the handling of extensive volumes of fine-grained annotations.

Using a Genetic Algorithm, Namdar et al. (43) recommended fine-tuning a qualified CNN for enhanced PCa diagnosis, resulting in an improved AUC. Furthermore, Kwon et al. (44) proposed a radiomics-based method for prostate image identification. The purpose was to identify multi-parametric MRI for clinically important PCa. Lay et al. (45) stated because MR imaging has its limitations, researchers suggest a different PCa detection technique that can be most effective. The cancer detection approach trains random ferns on MR sequences in the absence of one or more of these MR sequences and then uses these random ferns to add the MR sequences.

An approach for evaluating the grade for PCa has been suggested in this paper by Abraham and Nair (46). In this method, features are extracted utilizing deep network autoencoders in conjunction with hand-crafted features, subsequently categorized with a softmax classifier. Song et al. (47) have shown that radiologists manually mark the regions of significance for PCa and measure the scores for each area. The authors developed a model on patch-based DCNN that utilizes a combination of MRI data to distinguish between cancerous and non-cancerous patients of PCa.

According to Lemaitre et al. (48), new magnetic resonance imaging (MRI) approaches have emerged to enhance diagnostic accuracy. However, factors like observer variability and the visibility and complexity of lesions can still impact diagnosis. In this respect, CAD-based applications are designed to support radiologists in their clinical practice. Taking account of all MRI modalities, the authors suggest a CAD method. The goal of this CAD scheme was to detect the prostate position of cancer. Liu et al. (49) stated that for the classification of PCa, deep learning architecture was developed using the 3D multipara-metric MRI data. The Xmas-Net model was used for extracting features in this study. Mehrtash et al. (50) have demonstrated that to better detect PCa Computer-assisted diagnosis of MRI PCa may be used as a method of clinical decision support to help interpretation by radiologists. CNN models are used to detect the probability of a patient being affected or not.Yang et al. (51) provides an integrated method for detecting PCa that can simultaneously image PCa and locate lesions based on characteristics of the deep convolutionary neural network and SVM.

## 3 Transfer learning

**Figure 1:**
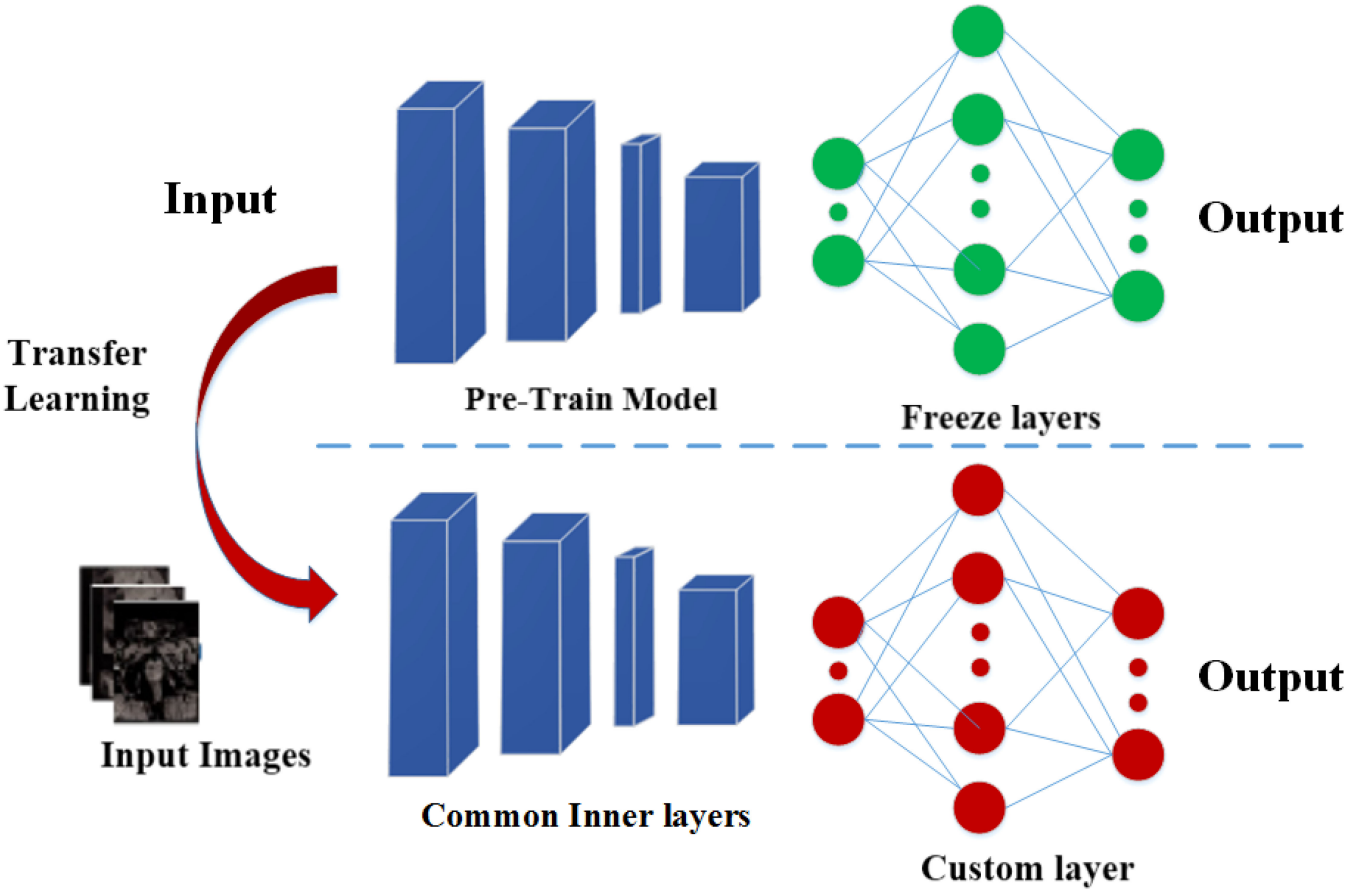
The top layers (last) are fine-tuned using TL.

TL is a technique for transferring information across domains Orenstein and Beijbom (52). Deep learning is a challenging and time-intensive process, especially in medical imaging, where a substantial amount of training data is needed to understand certain patterns. To address the challenge of limited data, medical imaging datasets are utilized to fine-tune the weights of deep learning models that were previously trained for different computer vision applications, thus accelerating the training process. The strategy frequently used in various computer vision problems is fine-tuning transfer learning. For classification, the dense layers are well-tuned, while the top layers are frozen. The proposed methodology for classifying prostate images using transfer learning is shown in **Figure 1**.

## 4 Materials and methods

The prostate dataset was obtained from the Cancer Imaging Archive Nolan (53). The dataset consists of prostate MRI images which are labeled with the help of radiologists. After performing minor data preprocessing, we carefully selected 221 cases of prostate cancer for our dataset. Our dataset comprised 98 cases of low-grade and 123 cases of high-grade cancer. Every patient included in the study exhibited an initial screening result that raised suspicions regarding prostate cancer. Subsequently, each of these patients underwent a biopsy, from which a GS was determined. These cases are annotated with two-class labels distinguishing between low-grade (GS = 3 + 4, 3 + 3) and high-grade (GS = 4 + 4, 4 + 3, 5 + 3, 3 + 5) cancer. For each case, T2w (sagittal and transaxial) and ADC images were provided to conduct experiments.

In our experimental setup, we adopted a systematic approach to partitioning the dataset to ensure robust training and evaluation of our model. We performed a random selection process, wherein 80% of the dataset was utilized for various purposes, including training and validation, while the remaining 20% of the images were exclusively designated as the test set. Within this 80% portion of the dataset, we further allocated distinct proportions for training and validation. Approximately 50% of the dataset was allocated for training, which served as the foundation for our transfer learning process. The remaining 30% of this 80% portion was dedicated to the validation set. This set played a pivotal role in monitoring the model’s performance during training. By periodically evaluating the model’s predictions on this validation subset, we could make informed decisions regarding hyperparameter tuning and model adjustments, ultimately ensuring that our model’s generalization capabilities were optimized. Lastly, the 20% of the images that constituted the test set were kept entirely separate from the training and validation data. This segregation ensured that our model was assessed on entirely unseen data, mirroring real-world scenarios where it would be applied to make predictions. The test set served to evaluate the model’s performance, providing a reliable measure of its ability to generalize to new and previously unseen data. Through this well-structured data partitioning strategy, we aimed to achieve a robust and thorough assessment of our model’s capabilities, while also upholding the principles of fairness, rigor, and transparency in our experimental approach.

### 4.1 Proposed approach

**Figure 2:**
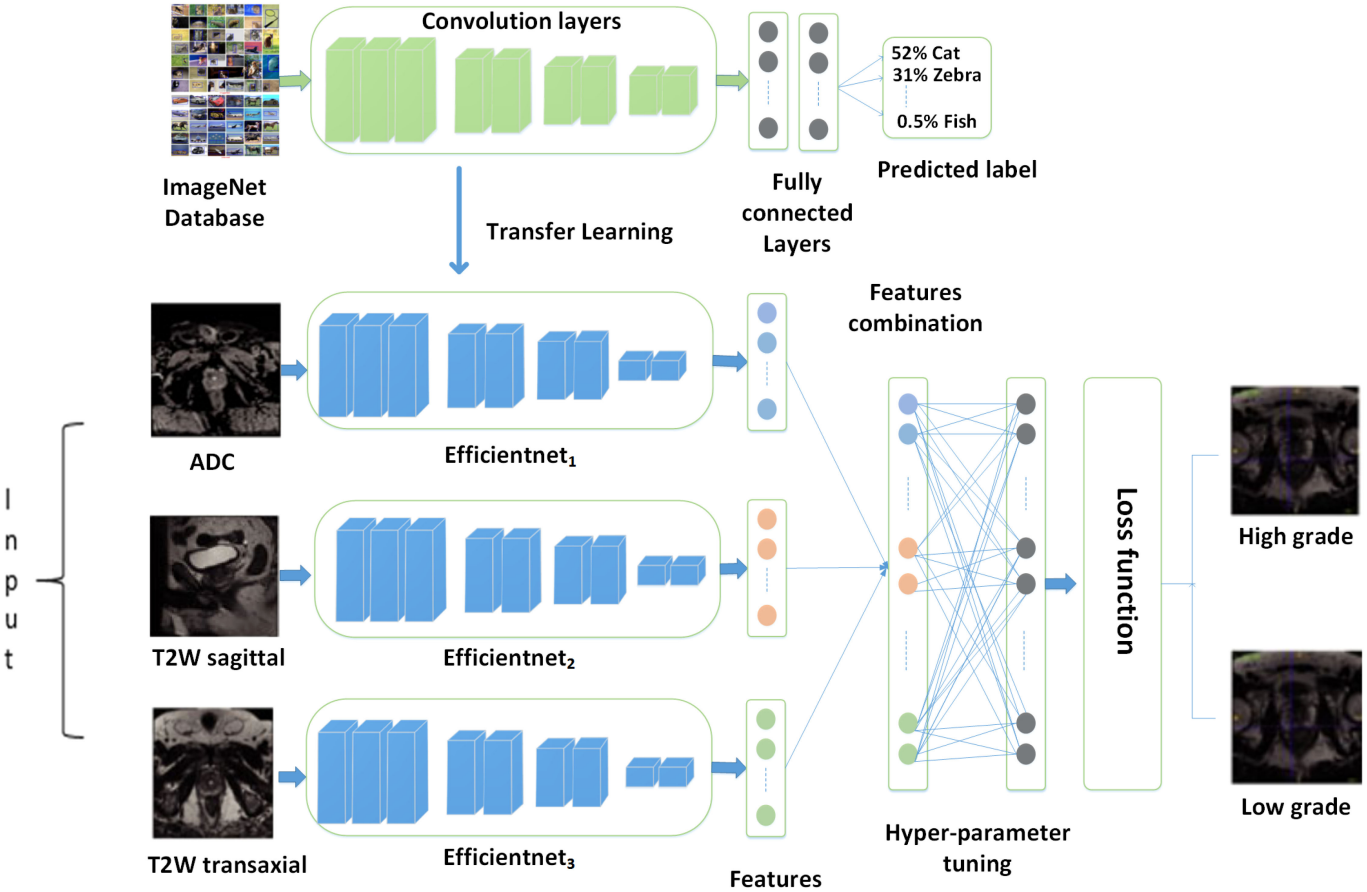
Proposed model.

This study presented a transfer learning model that utilizes multiparametric MRI for the classification of PCa into low-grade and high-grade. In **Figure 2**, the proposed model is mentioned. To learn features from multiparametric sequences (ADC, T2w), the authors make a transfer learning model with three branches and combine them to gain discriminative descriptors. A significant amount of training data is needed for deep convolution neural networks in medical imaging. When the available data is insufficient, deep CNNs often rely on pre-trained models. These models have been previously trained on extensive datasets, allowing for knowledge transfer, which is a fundamental aspect of TL.

**Figure 3:**
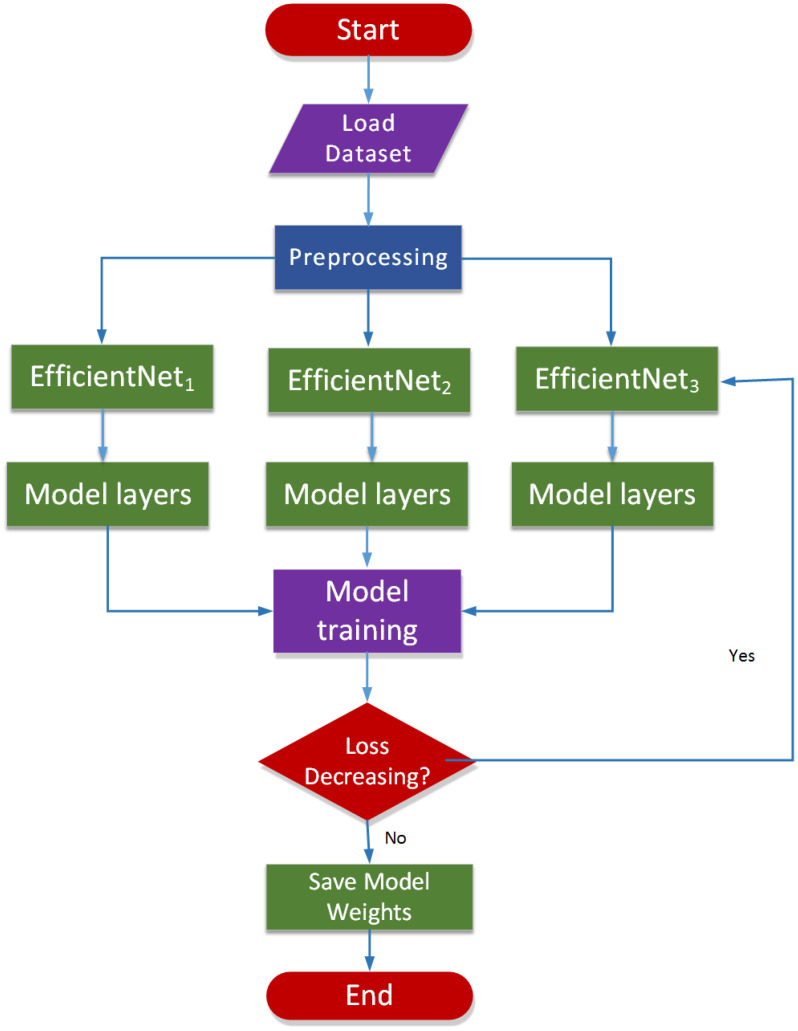
Workflow of the proposed model.

**Figure 3** describes the suggested model’s workflow. Transfer learning-based multi-parametric MRI model to automatic PCa identification is presented in this study. Various sequences of MRI reveal distinct aspects of PCa. T2-weighted (T2w) and ADC (Apparent Diffusion Coefficient) imaging modalities offer distinct insights, and their integration can significantly enhance the accuracy of PCa classification. To learn features from multi-parametric sequences T2w (sagittal and transaxial) and ADC, the authors make a transfer learning model with three branches of architecture to gain features separately for each modality and then combine them to gain one feature vector. We feed these sequences simultaneously in the network and their concatenation after the convolutional layer. Such a fusion approach allows the learning process to generate effective and discriminating PCa-related characteristics of multiple modalities mutually influenced by each other. To achieve better performance, we fine-tune our model by changing the top layers and defining the last layer classes to two nodes, as we identify PCa as a low-grade and high-grade form of cancer. After optimizing the MPTL methodology, we could perform the task of classifying prostate images. The performance of classifying PCa could be further improved by improving the ability to combine learned features.

### 4.2 Transfer learning using a single sequence to extract features

Due to the limited availability of prostate cancer data, we have opted for a transfer learning strategy instead of training an entire deep-learning neural network from the ground up. Specifically, we have harnessed the power of established deep learning architectures like ConvNets, AlexNet, and VGGNet, which have previously been trained on ImageNet datasets and are readily accessible as pre-trained networks. By implementing the EfficientNet architecture across multiple MRI series, we can extract essential features from ADC, T2w sagittal, and T2w transaxial images. This innovative approach allows us to transfer the knowledge acquired from ImageNet and effectively characterize PCa images.

### 4.3 A multisequence MRI-based feature fusion method

Different MRI modalities of PCa demonstrate different aspects. Various sequences of MRI disclose various PCa kinds. To provide separate and complementary data, T2w and ADC are recorded, and their combination can effectively increase the precision of PCa diagnosis. It is efficient to obtain the simultaneous information from MRI in deep learning method to optimize the relation between different MRIs. We feed these sequences simultaneously in the network and their concatenation after the convolutional layer. Such a fusion approach allows the learning process to generate effective and discriminating PCa-related characteristics of multiple modalities mutually influenced by each other. The performance is seen as the final joint characteristic after fully connected layers.

### 4.4 Developing a fine-tuned training strategy

**Figure 4:**
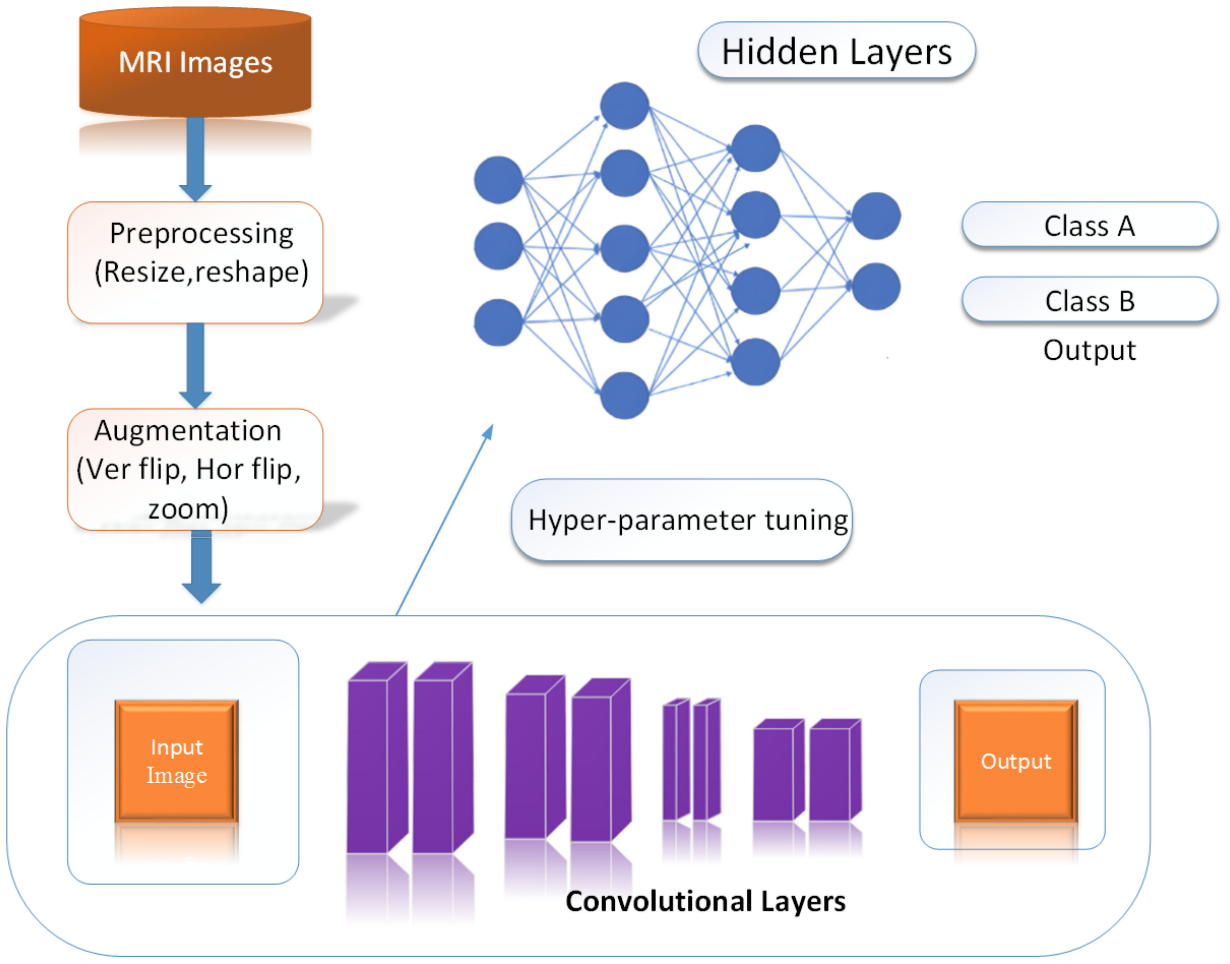
Model into work.

**Figure 5:**
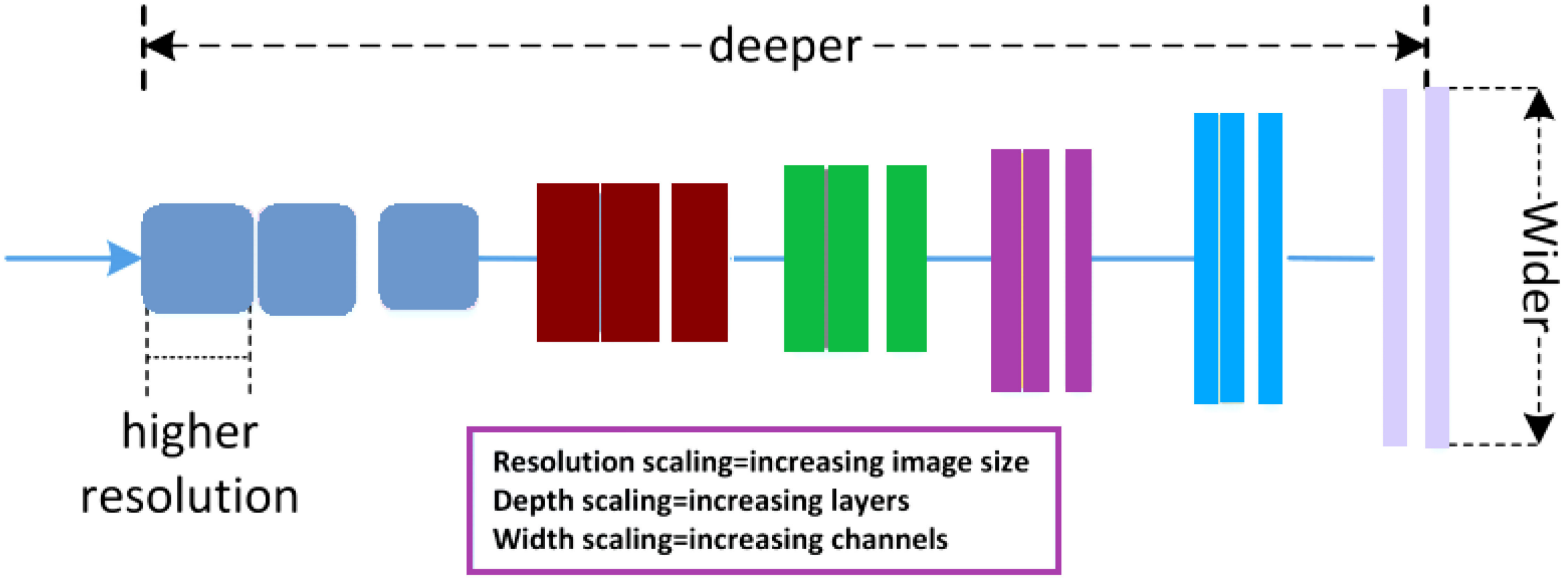
Efficient-net baseline model.

In our approach utilizing the Efficient-Net architecture, we took several steps to enhance the classification of PCa into high and low-grade. We integrated fully connected layers into the network and fine-tuned it using our dataset. This fine-tuning process was pivotal in adapting the model to our specific classification task. To boost the feature extraction capabilities of our model, we introduced custom layers, including global average pooling within the classification layers. This addition helped in capturing more nuanced features from the medical images, which is crucial in accurately classifying cancer. What sets our approach apart is the use of Efficient-Net architectures, which come with distinct advantages. These models are not only faster in classification, being 6.1× faster compared to existing CNN models, but they are also significantly smaller, being 8.4× smaller. Importantly, their compact size doesn’t compromise their accuracy. Our Efficient-Net models leveraged TL based on architectures pre-trained on the extensive ImageNet dataset, known for its high accuracy and efficiency. This TL approach allowed us to benefit from the knowledge embedded in these pre-trained models, especially when our own dataset was limited. To further enhance the training process and ensure robustness, we employed data augmentation. This technique plays a crucial role in augmenting the dataset, increasing the diversity of training samples, and consequently, improving the model’s ability to generalize to unseen data. It is particularly effective in preventing overfitting, a common challenge in classification tasks. One notable aspect of our strategy is the use of a pre-trained model as a feature extractor. In this approach, the last fully connected layer is removed, and the remaining layers are treated as a fixed feature extractor. This significantly accelerates the training process. In essence, our approach combines the advantages of Efficient-Net architectures, Transfer Learning, data augmentation, and a pre-trained feature extractor to enhance the classification of prostate cancer. **Figure 4** provides a visual representation of our model in action, demonstrating its potential in the field of medical image classification. The performance of the baseline Efficient-Net architecture is demonstrated in **Figure 5**.

### 4.5 Evaluation metrics

The output of input images is typically evaluated using the evaluation matrices listed below. The confusion matrix can be used to measure these matrices, including accuracy, precision, recall, and F1-score. The confusion matrix has four different types of parameters, where TP denotes a true positive, TN denotes a true negative, FP denotes a false positive, and FN denotes a false negative. as shown in **Table 1** and equations are given below.

**Table 1:** Literature review regarding PCa classification.

Author(s)	Description of Research	Methodology	Dataset Used	Evaluation Measures
Chenet al. (34)	Use transfer learning for prediction of PCa	InceptionV3, VGG-16	PROSTATEx	AUC, ROC
Xu et al. (35)	Utilize the residual networks for detecting PCa	Res-Net	PROSTATEx	ROC, HoM
Alkadi et al. (36)	Using deep convolutional encoder-decoder for detection	DCNN	I2CVB	Accuracy, DSC, IoU
Kwon et al. (44)	Apply CART and LASSO for detecting PCa	CART, LASSO	PROSTATEx	ROC, AUC
Lay et al. (45)	Apply Random ferns for classifying PCa	Random ferns	PROSTATEx	ROC, AUC
Abraham and Nair (46)	CNN inceptionV3 for feature pooling and selection	CNN, SVM	PROSTATEx	Accuracy, kappa, PPV
Songet al. (47)	Model with patchbased DCNN for PCa	DCNN	PROSTATEx	AUC
Lemaitre et al. (48)	RF classifier for feature learning and classification	RF classifier	I2CVB	AUC
Liu et al. (49)	Xmas-Net model used for extracting features	Xmas-Net	PROSTATEx	ROC, AUC
Mehrtash et al. (50)	Probability of patient being affected or not	CNN	PROSTATEx	ROC
Yanget al. (51)	The model with DCNN, SVM to detect PCa	DCNN, SVM	PROSTATEx	FROC, ROC, LLF, NL

True Positives: The precise predicted positive values demonstrate that the predicted and the actual class value are both positive.

True Negatives: These are the accurately predicted negative values, showing that both the predicted and the actual class value are negative.

False Positives: When the predicted class is true but the actual class is false.

False Negatives: When the predicted class is no but the actual class is yes.

Accuracy: Accuracy is the most used performance metric, which may be calculated as the ratio of correctly expected observations to all observations. Having high accuracy will lead one to believe that our models outperform.


(1) $$Accuracy=\frac{TP+TN}{TP+TN+FP+FN}$$

Precision: Precision is the ratio of accurately predicted positive observations to all predicted positive observations.


(2) $$Precision=\frac{TP}{TP+FP}$$

Recall: Recall is defined as the ratio of accurately predicted positive observations to all of the actual class observations.


(3) $$Recall=\frac{TP}{TP+FN}$$

F1 score: The weighted average of recall and accuracy is the Score. This score takes into consideration both false positives and false negatives. Although it is not as easy to immediately understand as accuracy, it is typically more beneficial than precision, especially if we have an uneven class distribution. If false positives and false negatives result in equal losses, accuracy performs better. It is simpler to include both accuracies and recall if the cost of false positives and negatives is significantly different.


(4) $$F1-Score=\frac{2\times Precision\;xRecall}{Precision+Recall}$$

### 4.6 Experimental settings

To get generalized results, the authors repeated the experiment several times, looking at different learning and test data combinations. To increase the robustness of the presented MPTL approach and to reduce overfitting, the authors used the data augmentation technique to increase the size of the training data of different image transformations. Before transferring images as input to the networks, the authors conducted some preprocessing steps. To fit the model, images are resized or reshaped from the original size to 244 × 244 for the family of Efficient-Net models for image classification. The Adam optimizer was used to further train the entire set of pre-trained Efficient-Net models.

The settings utilized to conduct the experiments are as follows.

• Experiment carried out using a Google Colab Pro platform with a GPU T4 P100 and 25 Gigabytes memory.
• Batch size of 16.
• Learning rate from le-1 to le-6 with weight decay of le-4.
• Epochs are set to 150.
• The Adam optimizer was used to further train the whole set of pre-trained Efficient-Net models Perez and Wang (54).

### 4.7 Experiments and results

We conducted an extensive comparative analysis to evaluate our proposed model alongside eight baseline methods. Initially, we employed a transfer learning model without fine-tuning, utilizing image features directly from ImageNet for experimentation. We then delved into three additional baseline experiments, finetuning single MRI sequences, specifically T2-weighted (T2w) and ADC (Apparent Diffusion Coefficient), for prostate cancer classification. Subsequently, we extended our experiments to three more baseline experiments, employing two MRI parameters as input. The comprehensive classification results for both our method and the eight baseline methods are meticulously detailed in **Table 2**.

**Table 2:** Comparison results of PCa classification.

References	Methods	Accuracy	Precision	Precision	F1-score
Chen et al. Chen et al. (34)	VGG-16	83	82.42	88.23	86.78
Kwon et al. Kwon et al. (44)	CART	82.0	81.84	81.46	79.61
Le et al. Le et al. (31)	ResNet	82.09	82.27	82.88	82.34
Muhammad Muhammad et al. (55)	inceptionV3	80.09	78.95	83.96	81.61
Serbanescu et al. Şerbănescuet al. (2)	GoogleNet	60.9	58.78	59.36	57.89
Present work (MPTL)	EfficientNet-B0	84.44	87.5	84.0	85.71
	EfficientNet-B5	86.67	83.33	90.90	86.95
	EfficientNet-B7	88.89	91.67	88.0	89.47

To assess the classification performance of our proposed MPTL model, we carried out a comprehensive evaluation, comparing it with state-of-the-art prostate cancer classification methods, including both deep learning and machine learning-based approaches. These comparisons were conducted using our prostate cancer datasets, and we followed the experiment settings outlined in these reference papers to ensure a fair and equitable assessment. **Table 3** meticulously presents the precision, recall, and accuracy metrics achieved by both our approach and the comparative methodologies. It’s noteworthy that deep learning-based techniques outperformed methods relying on traditional radiomics features or conventional machine learning approaches. This observation highlights the capability of deep learning-based techniques to capture more distinctive features for the identification of prostate cancer.

**Table 3:** Comparison results of PCa MRI.

	Accuracy	Precision	Recall	F1-score
MPTL-B0	84.44	87.5	84.0	85.71
T2w sagittal	71.43	72.13	73.79	72.66
T2w transaxial	73.78	71.43	79.39	75.44
ADC	74.72	73.86	78.30	76.06
ADC and T2w sagittal	81.81	82.97	86.67	84.78
ADC and T2w transaxial	83.33	85.1	83.34	84.20
Sagittal and T2w transaxial	82.21	86.95	83.33	85.10
MPTL-B5	86.67	83.33	90.90	86.95
MPTL-B7	88.89	91.67	88.0	89.47

Our method performs better at classification than the preceding approaches. This is due to the fact that convergence issues and over-fitting issues with little data on PCa also hinder deep network training. In comparison, the image details in the transfer learning model using Efficient-Net were considered by our MPTL model. Therefore, as compared to previous classification techniques, our technique evaluates more precise parameters for PCa and achieves more efficiency.

This method classified the input image into cancer types with low and high grades. We elaborate on the experimental results performance to distinguish between the aggressive and non-aggressive forms of cancer. The Efficient-Net B7 architecture, which was trained on images of PCa, produces the greatest results. **Table 3** shows the results of the proposed methods. In Şerbănescu et al. (2), authors apply the Google-Net approach for the identification of PCa classification for binary classification to distinguish low and high-grade forms of cancer and achieve 60.9 accuracies and performance. In Chen et al. (34) authors apply the VGG-16 approach for the identification of PCa classification for binary classification to distinguish the low and high-grade forms of cancer and achieve 83 accuracies and performance.

In Kwon et al. (44), authors apply the CART approach for the identification of PCa classification for binary classification to distinguish the low and high-grade forms of cancer and achieve 82.0 accuracies and performance. In Le et al. (31), authors apply the ResNet approach for the identification of PCa classification for binary classification to distinguish low and high-grade forms of cancer and achieve 82.09 accuracies and performance. In Muhammad et al. (55), authors apply the inceptionV3 approach for the identification of PCa classification for binary classification to distinguish the low and high-grade forms of cancer and achieve 80.09 accuracies and performance.

The comparison results of different methods with multi-parametric modalities are shown in **Table 2**. The results demonstrate that our proposed approach with a fusion of three modalities performs better results than single modalities and pair of modalities which depicts that our approach performance is better on multiple modalities.

**Figure 6:**
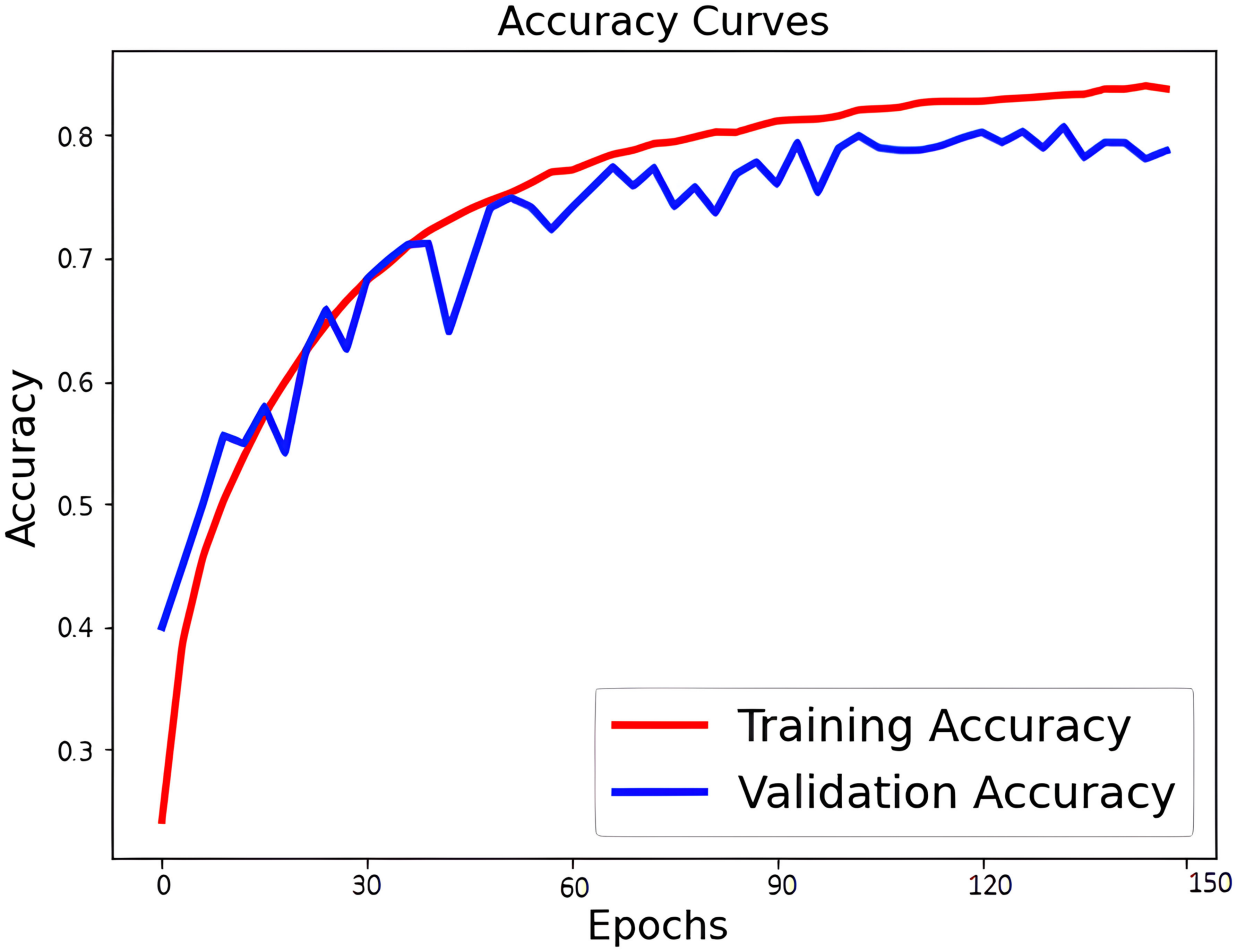
Accuracy curves of PCa classification.

**Figure 7:**
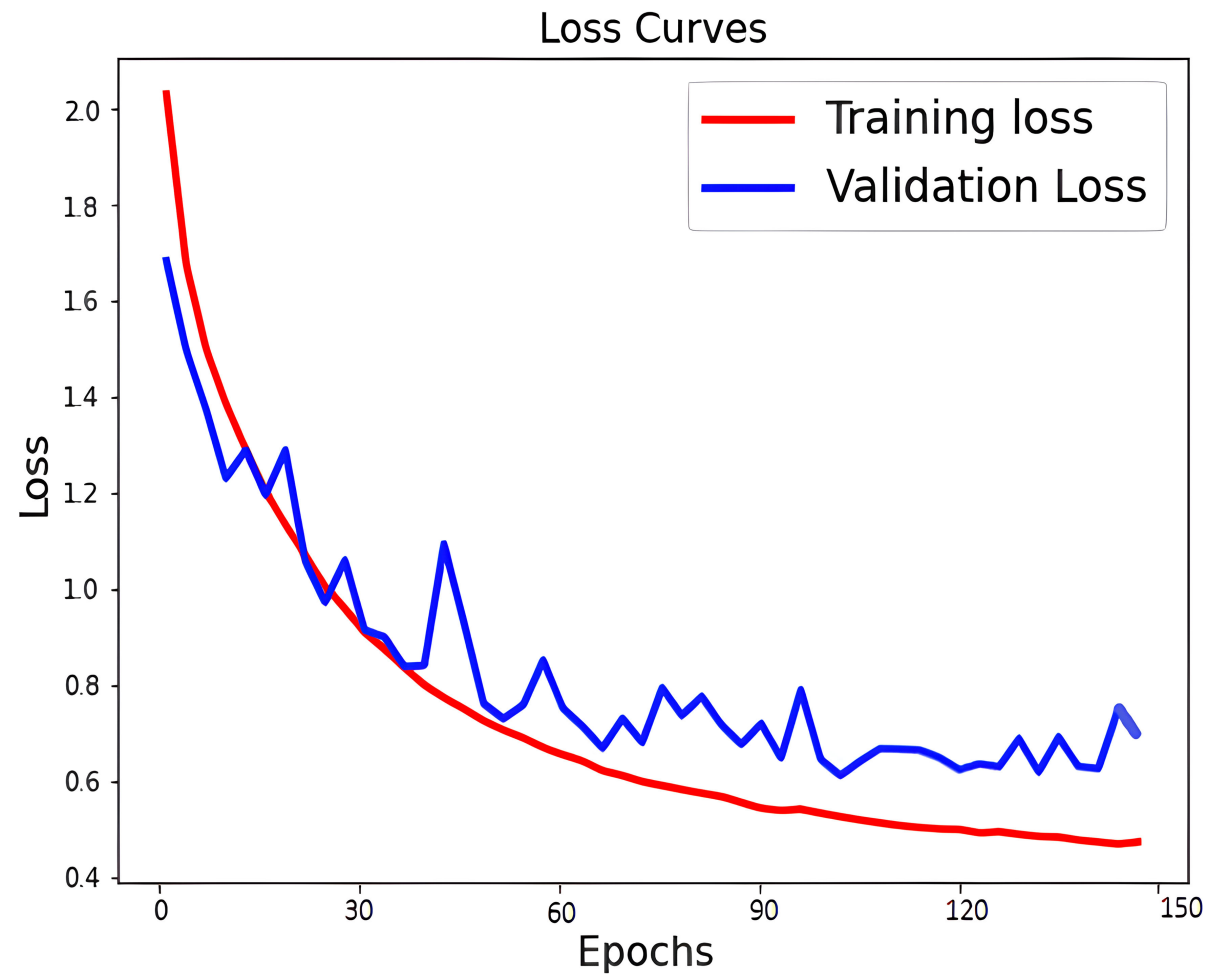
Loss curves of PCa classification.

**Figure 8:**
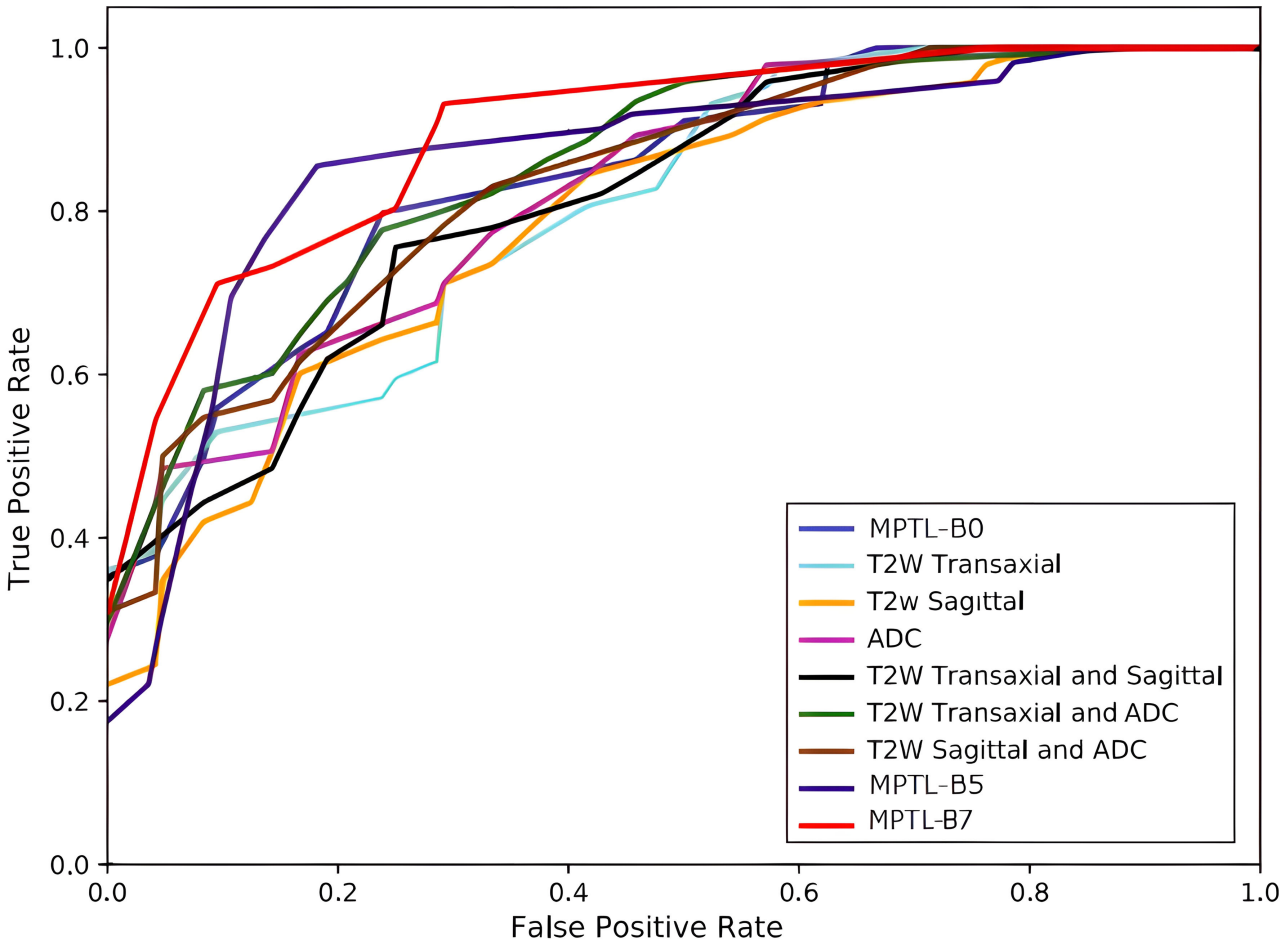
ROC curves of PCa.

The learning curve for accuracy and loss during training and validation is depicted in **Figures 6**, **7**. Our approach also shows better identification performance compared with the other machine learning approaches with extraction features from a single MRI sequence, showing that the methods based on deep learning will learn more high-level discriminative features. ROC curves of PCa classification are shown in **Figure 8**. The results demonstrate the performance of the model to identify input images is classified as low and high-grade forms of cancer that are aggressive and non-aggressive forms of cancer.

### 4.8 Cross dataset validation

To comprehensively evaluate the generalization performance of the proposed Multi-Parameter Transfer Learning (MPTL) model, an extensive experiment was conducted across diverse datasets. The primary objective of this experiment was to showcase the practicality and effectiveness of the MPTL framework in real-world scenarios where data sources may vary significantly.

For this purpose, we strategically leveraged two distinct datasets, both of which are publicly available to ensure transparency and reproducibility in our research. The first dataset, sourced from the Cancer Imaging Archive Nolan (53), is a substantial collection of medical images related to prostate cancer. The second dataset, referred to as I2CVB G. Lemaitre et al. (56), provides an additional set of prostate MRI images for comparative analysis. It’s worth noting that these datasets bring a wealth of diversity to the experiment. They exhibit variations in terms of image characteristics such as shapes, angles, sizes, resolutions, and formats. This diversity mirrors the real-world scenario where medical imaging data can originate from various sources and possess inherent dissimilarities. After minor cleaning, our training process was conducted on a robust training set comprising 5096 images from the first dataset. Subsequently, we rigorously assessed the model’s performance on an independent test set comprising 1371 images sourced from the I2CVB dataset. This demarcation of training and testing datasets enabled us to simulate a real-world scenario where a model is required to adapt and generalize across distinct data sources.

The results obtained in this cross-dataset experiment are highly encouraging. The proposed MPTL framework exhibited remarkable performance, further emphasizing its versatility and effectiveness in handling diverse data sources. Specifically, our model achieved an accuracy rate of 86.65%, indicating its capability to make correct classifications. The precision rate, measuring the model’s ability to correctly classify positive cases, stood at an impressive 83.36%. Furthermore, the recall rate, signifying the model’s capacity to identify all relevant instances, reached an impressive 89.18%. Lastly, the F1-score, which strikes a balance between precision and recall, demonstrated a robust performance at 86.13%. These outcomes underscore the generalization power of the MPTL framework for the classification of prostate MRI images. The model’s consistent and high-quality performance across datasets with diverse characteristics reinforces its potential utility in real-world medical applications, where data heterogeneity is often encountered.

## 5 Conclusion

In terms of replacing manual cancer assessment by radiologists using MRI images, CAD plays a critical role. There are, however, numerous risks and a high level of complexity involved in this task, along with expert-level opinions. The manual extraction of handcrafted features and subsequent classification not only consumes time but also introduces a higher likelihood of errors. To streamline the assessment process for radiologists and mitigate diagnostic errors, the necessity for an automated decision-making classification model becomes evident. In this paper, we introduce an innovative MPTL model for the automatic classification of PCa. Our model leverages knowledge from ImageNet to aid in the feature learning process from multi-parametric MRI (mp-MRI) sequences. These transferred features are combined to enhance the accuracy of PCa classification. A refined fine-tuning method including global average pooling is further applied to enhance PCa classification. As a result, the learned features exhibit significantly enhanced discriminative capabilities. Through an extensive series of comparative studies, we have highlighted the exceptional performance of our model in direct comparison to the prevailing state-of-the-art cancer classification methods. Our empirical results unequivocally establish the efficacy of our proposed approach in achieving high-precision PCa classification. Our findings highlight the potential benefits of transfer learning techniques from natural images to the medical domain, potentially offering valuable solutions in scenarios where the availability of annotated training datasets is limited for various practical considerations.

## Data availability statement

The raw data supporting the conclusions of this article will be made available by the authors, without undue reservation.

## Author contributions

Conceptualization, MM, MFM, and SA; methodology, MM, MSA, and MA; software, MM, KA, and MSA; validation, MFM, SA, and KA; formal analysis, MA and MSA; investigation, MSA, and SA; resources, MM, MFM and SA; data curation, KA, MSA, and MFM; writing—original draft preparation, MM, MFM, and SA; writing—review and editing, MSA, MA, KA, and MFM; visualization, MM, MFM, and SA; supervision, MFM and SA; project administration, MA and KA; funding acquisition, MA. All authors contributed to the article and approved the submitted version.
